# Comparison between Erigo tilt-table exercise and conventional physiotherapy exercises in acute stroke patients: a randomized trial

**DOI:** 10.1186/s40945-020-0075-2

**Published:** 2020-02-04

**Authors:** Suraj Kumar, Ramakant Yadav, Aafreen

**Affiliations:** 1 Department of Physiotherapy, UPUMS, Saifai, Etawah, UP 206130 India; 2 Department of Neurology, UPUMS, Saifai, Etawah, UP India

**Keywords:** Physiotherapy, Quality of life, Stroke, Tilt-table

## Abstract

**Background:**

Stroke is a common, serious, and disabling health-care problem throughout the world. Although great advances have been made in acute stroke management, the most of post-stroke care to reduce a patient’s dependency relies on rehabilitation.

**Purpose:**

To compare the effectiveness of exercises using an Erigo tilt-table and conventional physiotherapy in the rehabilitation of acute stroke patients.

**Methods:**

A total of 110 acute stroke patients (age 51.08 ± 7.48 years, 8.69 ± 4.62 days after stroke) were assigned randomly into two groups, 55 in each for 30 days of conventional physiotherapy (Group A) or Erigo tilt-table (Group B) rehabilitation. The National Institutes of Health Stroke Scale (NIHSS), Mini-Mental Scale Examination (MMSE), Modified Ashworth Scale were used to measure muscle tone, quality of life (QOL) and muscle strength (MMT), Affected upper (UE) and lower limb (LE) outcomes were assessed at baseline (day 0), after day 30 of the intervention and on 90th day of follow up. Repeated measures ANOVA followed by a Bonferroni post-hoc test and independent Student’s t-test were used for statistical analysis to evaluate the improvement in outcome variables within and between the groups.

**Results:**

Both the treatments were effective. Notably, Group B patients showed a significant improvement in both QOL (*p* < 0.001) and lower limb strength (*p* = 0.030) at day 90 and muscle tone (*p* = 0.011) at day 30 compared to Group A.

**Conclusion:**

Both the groups improved with time but the Erigo tilt-table group experienced greater improvement in QOL, NIHSS and muscle strength of the lower limb. Thus, Erigo tilt-table can be used for early rehabilitation of acute hemiplegic patients and improving their quality of life and motor system, resulting in better functional performances.

## Introduction

Stroke is a major cause of death and disability [1], and is often associated with negative secondary complications that might postpone or prevent recovery [2]. Early mobilization averts such negative effects and promotes recovery [3, 4]. However, it is challenging as many patients are severely impaired and are non-cooperative. Mobilization using an Erigo tilt-table is a well-tolerated method of mobilization and can be considered a safe system of early mobilization of patients with severe brain injuries and stroke [5, 6]. Patients in vegetative or minimally conscious state tolerate greater degrees of head-up tilt better with simultaneous leg movement [7]. Blood pressures and heart rate can be stabilized better with treatment with passive leg movements [8]. Moreover, an intensive cyclic leg loading verticalization protocol, started in the acute stages of a severe acquired brain injury, improves the short-term and long-term functional and neurological outcome of patients with disorder of consciousness [9].

The Erigo FES (Functional Electrical Stimulation) is fully synchronized with robotic leg movements. Thus, the cyclic FES to the leg muscles can effectively induce venous return, thereby helping to improve cerebral blood flow and to maintain the blood pressure of individuals with spinal cord injury under orthostatic stress [10, 11].

Neuro-rehabilitation plays a central role in successfully reducing the long-term effects of stroke and achieving optimal functional recovery for community re-integration. Neuroplasticity is the basic mechanism underlying improvement in functional outcome after stroke [12]. Therefore, an important goal of rehabilitation of stroke patients is the effective use of neuroplasticity for functional recovery. Thus, high-dose intensive training and repetitive practice of specific functional tasks are important for recovery after stroke.

The electromechanical/robotic devices that ensure automation of lower limb movements during locomotion were developed to help the physiotherapists by increasing the safety, intensity, and standardization of non-robotic body weight supported treadmill training, generate complex multisensory stimulation, offer extensive extrinsic biofeedback to the patient, and reduce the working costs [13].

The robotic Erigo tilt-table consists of a stretcher that can be tilted between 0°-80° and footplates with integrated springs for leg loading. Footplates do stepping-like movements. Training on the Erigo combines mobilization out of bed, body verticalization, and rhythmic leg movement with cyclic loading thus contributing to the prophylaxis of secondary complications caused by a prolonged period of immobility. The benefits offered by the Erigo compared to mobilization using a conventional tilt table are as follows:

- Passive movement of legs combined with verticalization of the patient
- A load is alternately applied and removed from the patient’s legs
- The leg movement can be adapted separately for the patient’s right and left leg according to his needs ([14], Erigo User Manual Part-1).

The most important advantage of using robot technology in rehabilitation is to deliver high-dosage and high-intensity training.

Prolonged inactivity leads to deconditioning and complications appear during the first days of bed rest and worsen the devastating neurological injury. Therefore, early intervention by rehabilitation protocols should begin, to reduce the prolonged bed rest complications, stimulate the afferent sensory system and to reduce spasticity. Although earlier studies on rehabilitation with robotics proved to be beneficial, there are very few clinical studies available on acute stroke patients and its comparison with conventional physiotherapy. Thus, this study hypothesized that Erigo tilt-table therapy will be more effective than conventional physiotherapy for early mobilization and rehabilitation in acute stroke patients.

## Methods and materials

### Subjects

The participants included in this study are post-stroke hemiparesis subjects referred to the Department of Neurology, ‘X’ aged 30–60 years either male or female diagnosed clinically by a neurologist with ischemic or hemorrhagic stroke, within 7–28 days of onset and with scores on the National Institutes of Health Stroke Scale (NIHSS) between 11 and 22. The present study has the approval of the Institutional Local Ethical Committee and informed consent was obtained from all the participants so that patients understood the purpose of the study. Subjects were excluded from the study if they had a metal implant, recurrent stroke, chronic renal failure, cognitive and speech problem, hemiplegia due to non-vascular causes (malignancy, infections, tumours, brain injury, etc.), sensation loss in the lower extremity, or poor sitting balance.

### Procedure

The subjects were randomized into two groups by lottery method [15], Group A for conventional physiotherapy and Group B for robotic Erigo tilt-table rehabilitation. One hundred forty folded papers of the same shape and size were marked either Group A (70) or Group B (70) and were kept in a box. Paper drawn by the patient allocated the mode of treatment. The allocation was concealed and the assessor was blinded to group allocation. After group allocations, respective subjects’ were treated either with conventional physiotherapy or robotic tilt- table therapy. Both treatments were given as individual treatment by the same physiotherapist for 30 regular days (except Sundays) and reassessment was done after 30 days and on follow up at 90th day. The duration of each treatment session was about 50 to 60 min per day. The demographic characteristics such as age, weight, height, BMI (body mass index), the side affected, and the onset of stroke (duration) of the two treatment groups were assessed at baseline before randomization. Similarly, outcome variables such as muscle strength (upper limb and lower limb), NIHSS, Ashworth, MMSE and QOL were also assessed by same tester and same physiotherapist supervising the test procedure at baseline (day 0) as well as at the end of treatment (day 30) and on follow- up (day 90). During 30–90 days, subjects were asked to perform a home-exercise program. All the patients were provided with the home-exercise booklet which includes written instructions and figures. Subjects were once asked to perform these exercises under the supervision of physiotherapists and then to continue at home on their own or with the help of attendants. During this period the patients were contacted every 15 days either at the hospital or telephonically to determine whether they are performing the exercises properly or not.

Test and re-test of two groups were conducted in the same place in the same environment. Before experimentation, all subjects were well taught about the measurement variables and their outcomes. The subjects were also informed about experimental risks if any. All subjects were allowed to take treatment for their comorbid conditions such as hypertension, dyslipidemia, hypothyroidism, the cardiac problem in both the conditions under the supervision of neurologists. No other treatment was allowed other than those mentioned above.

### Training protocols

#### Conventional physiotherapy (Group A) [16]

All the exercises were done for 10 repetitions, 2 sets with 10 s hold once a day under the supervision of the physiotherapist which includes the following:

- Full range of motion (ROM) exercises – passive and active-assisted range of motion exercises for the upper limb including shoulder (flexion, extension, abduction and adduction), elbow (flexion and extension), forearm (supination and pronation), wrist (flexion, extension, radial and ulnar deviation), and for the lower limb including hip (flexion, extension, abduction and adduction), knee (flexion and extension), ankle (dorsiflexion, plantar flexion, eversion and inversion).
- For spasticity management - Positioning of the limb, prolonged icing, brushing, gentle stroking, and gentle tapping [17–19]
- Common mat activities including turning from supine to side-lying to prone and vice versa, prone to prone on an elbow, prone on elbow to prone on hand; prone on hand to quadruped; quadruped to kneeling; kneeling to half-kneeling; half kneeling to standing with support; standing with support to the standing without support.
- Bridging exercises.
- Prolonged and gradually progressive stretching of hamstrings, calf, and wrist.
- Strengthening exercises included isometrics of the back, quadriceps, and gripping exercises.
- Gentle and controlled weight-bearing exercises.
- Balance and coordination exercises.

#### Erigo tilt-table therapy (Group B)

Erigo tilt-table therapy was administered according to the following protocol. The patient received a treatment session of 40 min, 6 times per week [20] for about 4 weeks [21] (Table 1).

**Table 1:** The exercise protocol on Robotic Erigo tilt-table

Phase I (1st week)	At 30° angle for 40 min with 1-min hold after every 12 min at 0° angle
Phase II (2nd and 3rd weeks)	At 50° angle for 40 min with 1-min hold after every 12 min at 0° angle
Phase III (4th week)	At 75° angle for 40 min with 1-min hold after every 12 min at 0° angle

The Robotic tilt table exercise session was followed by a 15 min exercise program for upper extremities which including range of motion, strengthening and stretching exercises of shoulder, elbow, wrist, and fingers.

No specific gait training was administered to both the groups, but ambulation was offered to all patients when they were able to ambulate.

### Outcome variables

The assessment of QOL was done according to the SF-36 assessment tool [22]. It is a multipurpose, self-administered, short-form (SF) health survey with 36 questions that measure generic health status in the general population. These questions consist of physical functioning, role functioning (physical and emotional), body pain, general health, vitality, social functioning, and, mental health. Response choices are numbered from left to right, starting with 1. The maximum scores obtained from 36 questions where 149 which represents best QOL whereas minimum score 36 represents the worst [23]. The scale was used earlier in acute stroke patients [24, 25].

Muscle strength was measured by the MRC (Medical Research Council) classification of Manual Muscle Testing (MMT). Overall upper limb and lower limb muscle strength were recorded. For the upper limb, MMT was performed for shoulder flexion, shoulder extension, shoulder abduction, shoulder adduction, shoulder external rotation, shoulder internal rotation, elbow flexion, elbow extension, forearm pronation, forearm supination, wrist palmar flexion, wrist dorsal flexion, ulnar deviation, and radial deviation. The average score of these measures was considered as overall upper limb muscle strength. Similarly for the lower limb, MMT was done for hip flexion, hip extension, hip abduction, hip adduction, hip external rotation, hip internal rotation, knee flexion, knee flexion with leg external rotation, knee flexion with leg internal rotation, knee extension, ankle plantar flexion, ankle plantar flexion (soleus), foot dorsiflexion and inversion, foot inversion, foot eversion with plantar flexion and foot eversion with dorsiflexion. The average score of these measures was recorded as overall lower limb muscle strength. The positioning of the patients for performing MMT was according to standard norms and was also used earlier in a published study [26, 27].

The National Institutes of Health Stroke Scale or NIH Stroke Scale (NIHSS) is a tool used by healthcare providers to objectively quantify the impairment caused by a stroke. The NIHSS is composed of 11 items, each of which scores a specific ability between a 0 and 4. For each item, a score of 0 typically indicates a normal function in that specific ability, while a higher score is indicative of some level of impairment. The individual scores from each item are summed to calculate a patient’s total NIHSS score. The greatest possible score is 42, with the lowest score being 0 [28].

The Mini-Mental State Examination (MMSE) is a 30-point questionnaire used extensively in clinical and research settings to measure cognitive impairment. Administration of the test takes between 5 and 10 min and examines function including registration (repeating named prompts), attention and calculation, recall, language, ability to follow simple commands and orientation. Any score greater than or equal to 24 points (out of 30) indicates a normal cognition. Below this, scores can indicate severe (≤9 points), moderate (10–18 points) or mild (19–23 points) cognitive impairment [29].

The Modified Ashworth Scale is a 6-point rating scale measuring muscle tone with ratings from 0 indicating no increase in tone to 5 indicating limb rigid in flexion or extension [30]. For the upper limb, the scores of shoulder adductors, elbow flexors, and wrist flexors were recorded and for the lower limb scores of hip adductors, knee extensors and ankle plantar flexors were recorded. The average of these readings considered as the global score [31].

### Statistical analysis

Continuous data were summarized by mean and standard deviation (SD) whereas discrete (categorical) data were described by number (n) and percentage (%). Two independent groups compared by independent Student’s t-test. Groups were also compared by repeated measures two factors (Time*Group) analysis of variance (ANOVA) and the significance of mean difference within (intra) and between (inter) the groups were calculated by Bonferroni post hoc test for multiple contrasts. Categorical variables were compared by the chi-square (χ^2^) test. A two-tailed (*α* = 2) *p* < 0.05 was considered statistically significant.

## Results

**Fig. 1:**
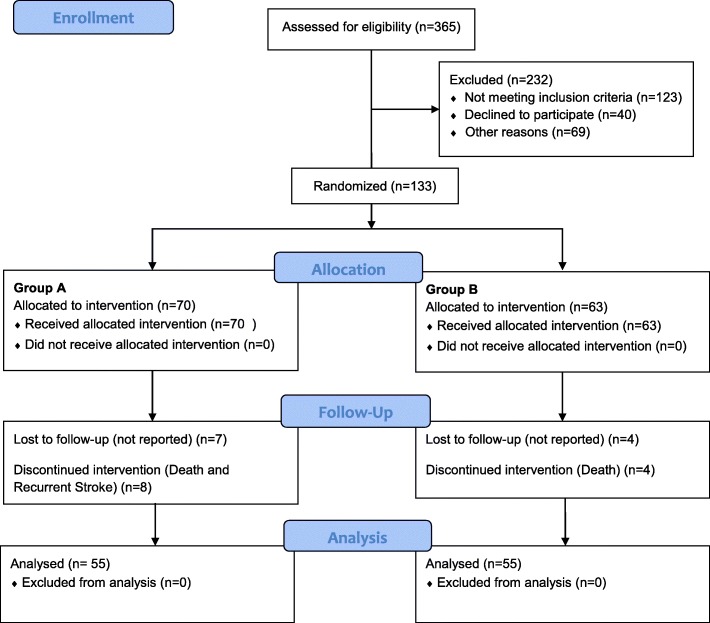
The flow diagram shows the number of patients in the treatment groups

A total of 133 patients were assigned out of 365 patients enrolled from the Department of Neurology from May 2017 to September 2018. Twenty-three patients dropped out for different reasons (see Fig. 1). Of these 110 patients who completed the study, 55 were treated with conventional therapy (Group A) and 55 with Erigo tilt-table (Group B) presented in Fig. 1. The mean age of the 110 patients was 51.08 ± 7.48 years. In 54 patients, stroke affected the right hemisphere. Patient demographic parameters are presented in Table 2. The training was started on average 8.69 ± 4.62 days after the stroke.

**Table 2:** Demographic characteristics (Mean ± SD) of two groups

Demographic characteristics	Group A (*n* = 55) (%) (mean +/− SD)	Group B (*n* = 55) (%) (mean +/− SD)	*P* value
Age (yrs)	51.36 ± 8.10	50.80 ± 6.86	0.695
Sex:			
Male	30 (54.5)	30 (54.5)	1.000
Female	25 (45.5)	25 (45.5)	
Weight (kg)^a^	65.62 ± 10.13	63.93 ± 7.14	0.314
Height (m)^a^	1.61 ± 0.09	1.60 ± 0.09	0.809
BMI (kg/m2)^a^	25.40 ± 3.72	24.89 ± 2.46	0.395
Side affected:			
Right	31 (56.4)	25 (45.5)	0.252
Left	24 (43.6)	30 (54.5)	
Lesion:			
Haemorrhagic	33 (60.0)	27 (49.1)	0.251
Ischemic	22 (40.0)	28 (50.9)	
SBP (mmHg)^a^	136.18 ± 16.50	132.18 ± 15.95	0.199
DBP (mmHg)^a^	86.91 ± 9.60	83.82 ± 8.92	0.083

*Group A* Conventional physiotherapy, *Group B* Erigo tilt-table, *BMI* Body mass index, *SBP* Systolic blood pressure, *DBP* Diastolic blood pressure

^a^ mean

### Demographic characteristics

The baseline demographic characteristics of the two groups are summarised in Table 2. On comparing, the baseline demographic characteristics were found similar (*p* > 0.05) between the two groups (Table 2) suggesting that both the groups were demographically matched and the outcomes would not be influenced by these parameters.

### Outcome measures

**Fig. 2:**
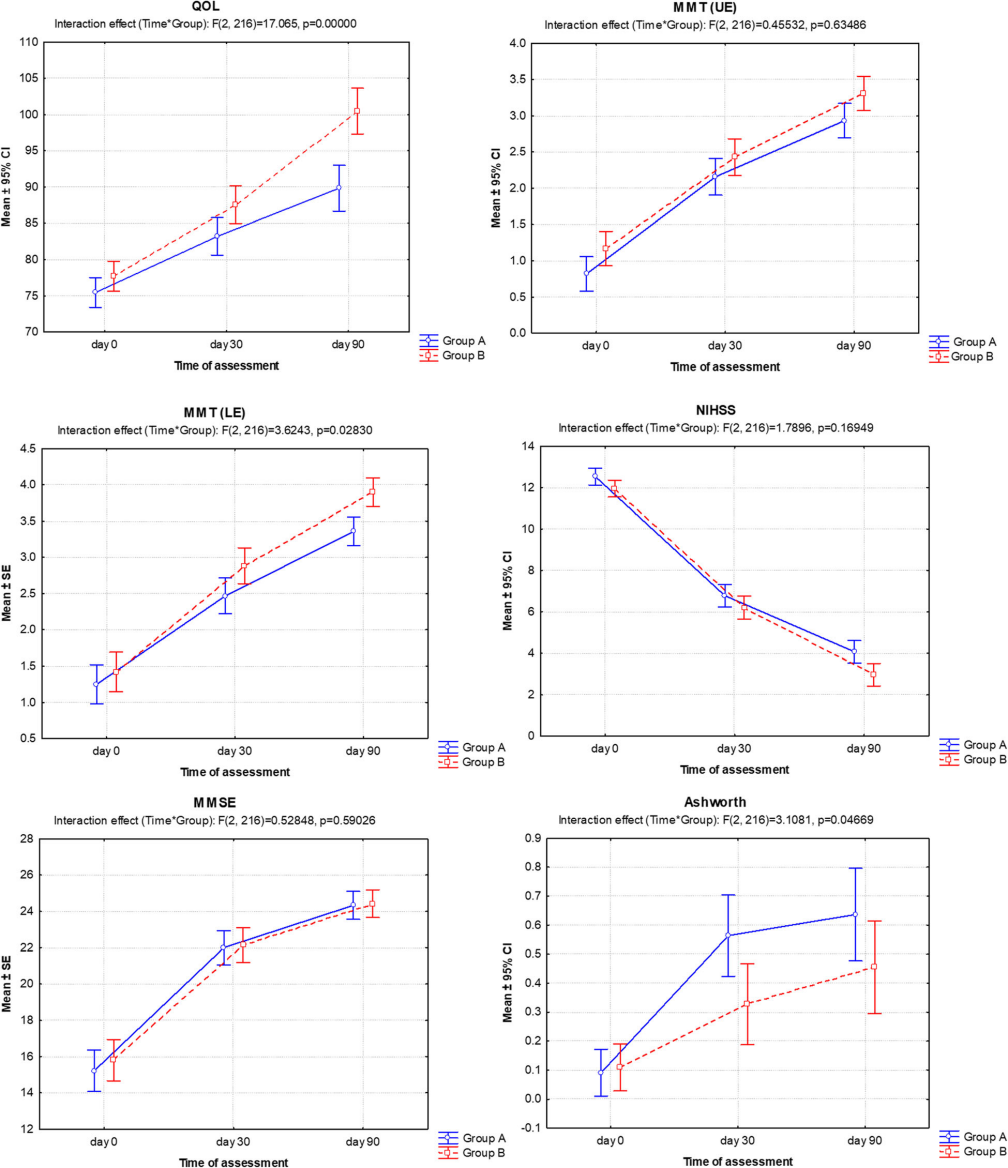
Line graphs showing the mean outcome measure score of two groups over the periods with an interaction effect. Vertical bars denote 95% confidence interval (CI). Group A: Conventional physiotherapy, Group B: Erigo tilt-table

The pre and post outcome measures of two groups is summarised in Table 3 and also depicted in Fig. 2. Comparison of the outcome measures of two groups over the periods (time), using ANOVA showed significant effect of groups on QOL (*F* = 11.77, *p* = 0.001), MMT (UE) (*F* = 4.36, *p* = 0.039), MMT (LE) (*F* = 6.15, *p* = 0.015) and NIHSS (*F* = 6.05, *p* = 0.015), and also periods on QOL (*F* = 310.36, *p* < 0.001), MMT (UE) (*F* = 701.26, *p* < 0.001), MMT (LE) (*F* = 546.51, *p* < 0.001), NIHSS (*F* = 1517.30, *p* < 0.001), MMSE (*F* = 617.73, *p* < 0.001) and Ashworth (*F* = 37.31, *p* < 0.001). Furthermore the interaction effect of both groups and periods (Groups*Time) on QOL (*F* = 17.06, *p* < 0.001), MMT (LE) (*F* = 3.62, *p* = 0.028) and Ashworth (*F* = 3.11, *p* = 0.047) were also found significant (Fig. 2).

**Table 3:** Outcome measure score (Mean ± SD) of two groups over the periods

Outcome measure	Day 0	Day 30	Day 90
QOL:			
Group A	75.45 ± 6.59	83.20 ± 9.41^a^	89.84 ± 11.74^ab^
Group B	77.71 ± 8.69	87.58 ± 9.93^a^	100.47 ± 11.97^ab^
*P* value^#^	1.000	0.321	< 0.001
MMT (UE):			
Group A	0.82 ± 0.90	2.16 ± 0.96^a^	2.93 ± 0.88^ab^
Group B	1.17 ± 0.86	2.42 ± 0.93^a^	3.31 ± 0.90^ab^
*P* value^#^	0.658	1.000	0.463
MMT (LE):			
Group A	1.25 ± 1.04	2.47 ± 1.01^a^	3.36 ± 0.89^ab^
Group B	1.42 ± 0.98	2.88 ± 0.83^a^	3.90 ± 0.54^ab^
*P* value^#^	1.000	0.241	0.030
NIHSS:			
Group A	12.53 ± 1.59	6.78 ± 2.11^a^	4.07 ± 2.07^ab^
Group B	11.95 ± 1.45	6.20 ± 2.00^a^	2.96 ± 1.99^ab^
*P* value^#^	1.000	1.000	0.035
MMSE:			
Group A	15.22 ± 4.45	22.00 ± 3.59^a^	24.33 ± 2.93^ab^
Group B	15.80 ± 4.10	22.15 ± 3.46^a^	24.42 ± 2.81^ab^
*P* value^#^	1.000	1.000	1.000
Ashworth:			
Group A	0.09 ± 0.29	0.56 ± 0.54^a^	0.64 ± 0.62^ab^
Group B	0.11 ± 0.31	0.33 ± 0.51^a^	0.45 ± 0.57^b^
*P* value^#^	1.000	0.181	0.793

*Group A* Conventional physiotherapy, *Group B* Erigo tilt-table, *QOL* Quality of life, *MMT (UE)* Manual muscle testing (upper extremity) or overall upper limb strength, *MMT (LE)* Manual muscle testing (lower extremity) or overall lower limb strength, *NIHSS* National institute of health stroke scale, *MMSE* Mini-mental state examination, *Ashworth* Ashworth scale-6

^a^*p* < 0.001- as compared to day 0 and ^b^*p* < 0.001- as compared to day 30 (intragroup comparison), ^#^(intergroup comparison)

Further, for each outcome measure, comparing the difference in mean across the periods (intragroup), Bonferroni test showed significant (*p* < 0.001) increase in QOL, MMT (UE), MMT (LE), MMSE and Ashworth while significant (*p* < 0.001) decreases in NIHSS at both day 30 and day 90 were noted compared to day 0 in both groups (except Ashworth in Group B) (Table 3). Furthermore, in both groups, QOL, MMT (UE), MMT (LE) and MMSE also showed significant (*p* < 0.001) increase while NIHSS showed significant (*p* < 0.001) decrease at day 90 as compared to day 30.

Similarly, for each outcome measure, comparing the mean between the groups (intergroup), the Bonferroni test showed similar (*p* > 0.05) mean between the groups at day 0 indicating all outcome measures comparable (Table 3). However, at day 90, both QOL (*p* < 0.001) and MMT (LE) (*p* = 0.030) were found significantly different and higher in Group B as compared to Group A. In contrast, at day 90, NIHSS was significantly lower (*p* = 0.035) in Group B compared to Group A.

### Net improvement

To determine the efficacy of one group over the other, the pre -to post- change in outcome measures were done and compared by independent Student’s t-test and summarised in Table 4. Analysis using Student’s t-test showed significant improvement in both QOL (*p* < 0.001) and MMT (LE) (*p* = 0.030) of Group B as compared to Group A at day 90. Furthermore on day 30, muscle tone (i.e. Ashworth scale) also improved significantly (*p* = 0.011) in Group B as compared to Group A. However, other parameters did not (*p* > 0.05) show significant improvement and were found to be statistically the same between groups.

**Table 4:** Pre-treatment to post-treatment change in outcome measure score (Mean ± SD) between two groups at two different periods

Outcome measure	Group A (*n* = 55)	Group B (*n* = 55)	*P* value
QOL:			
Day 30	7.75 ± 6.03	9.87 ± 6.84	0.086
Day 90	14.38 ± 8.83	22.76 ± 10.52	< 0.001
MMT (UE):			
Day 30	1.34 ± 0.54	1.26 ± 0.46	0.410
Day 90	2.11 ± 0.76	2.14 ± 0.65	0.851
MMT (LE):			
Day 30	1.22 ± 0.74	1.46 ± 0.69	0.074
Day 90	2.11 ± 0.90	2.48 ± 0.86	0.030
NIHSS:			
Day 30	−5.75 ± 1.95	−5.75 ± 1.89	1.000
Day 90	−8.45 ± 1.89	−8.98 ± 1.62	0.119
MMSE:			
Day 30	6.78 ± 3.26	6.35 ± 2.70	0.446
Day 90	9.11 ± 3.44	8.62 ± 2.93	0.422
Ashworh:			
Day 30	0.47 ± 0.57	0.22 ± 0.46	0.011
Day 90	0.55 ± 0.69	0.35 ± 0.58	0.104

*Group A* Conventional physiotherapy, *Group B* Erigo tilt-table, *QOL* Quality of life, *MMT (UE)* Manual muscle testing (upper extremity) or overall upper limb strength, *MMT (LE)* Manual muscle testing (lower extremity) or overall lower limb strength, *NIHSS* National institute of health stroke scale, *MMSE* Mini-mental state examination, *Ashworth* Ashworth scale-6

## Discussion

The study hypothesized that Erigo tilt-table therapy would be more effective than conventional physiotherapy for early mobilization and rehabilitation in acute stroke patients. This hypothesis was found to be partially true as outcomes measures, QOL, NIHSS, muscle strength lower limb improved more in Group B. The improvement could be related to the fact that robotic tilt-table rehabilitation may offer standardized, intensive and repetitive exercises, proper body weight support, with an appropriate sensory feedback amount and a controlled progressive verticalization. It was also found in another study that ERIGO training could be a valuable tool for the adaptation to the vertical position with a better global function improvement, as also suggested by the sensory-motor and vestibular system plasticity induction in post-stroke patients [14].

One study suggested that robotic verticalization maximizes the potential for longitudinal weight-bearing through the lower extremities in a position of hip-extension/knee-extension/ankle-dorsiflexion, which is difficultly obtained in the physiotherapy verticalization setting. Moreover, robotic verticalization allows strengthening exercises using body weight shifting from one leg to the other, which is not easily carried out by severe post-stroke patients [14]. Some studies found that greater cerebral blood flow modulation during robotic verticalization in comparison to physiotherapy verticalization could further support plastic changes within sensory-motor areas and vestibular system, with the consequent motor and cognitive function amelioration [32, 33].

Rehabilitation on a tilt-table has been reported to be a useful way to mobilize severely impaired or non-cooperating patients, since it improves circulation, prevents contractures, and increases pulmonary ventilation [34, 35]. Our study supports the safety and effectiveness of robotic Erigo tilt table verticalization in bedridden post-stroke patients even in the acute phase.

Our study suggests that a reduction in motor impairments is greater after tilt-table exercises as it reduces long-term spasticity and improves strength. Our study supports another study that showed a significant increase in the EMG patterns of the extensors and flexors of the affected leg muscles during flexion and extension movements of both legs and clinical scores in patients undergoing the progressive task-oriented training on the tilt table compared to the other groups [36].

In our study, the Ashworth scores were less in the tilt-table group when compared with conventional physiotherapy group initially after 30 days of intervention which shows that tilt-table therapy reduced spasticity. Another study has also showed that extensor spasms are reduced after tilt table standing [37]. Robot-based rehabilitation has been shown to improve motor performance by boosting brain plasticity [38, 39].

With respect to the hemodynamic effects of robotic verticalization, it has been shown that robotic tilt-tables are effective in preventing blood pressure drops [40], which may prevent orthostatic responses to verticalization by improving venous return and, hence, potentiating the cardiac output and the cerebral blood flow. In addition to improving orthostatic tolerance, robotic tilt-table may also be an effective exercise tool in initiating the rehabilitation process at the earliest possible time point after stroke may minimize the reduction in aerobic fitness that occurs due to inactivity. It was also found that robotic tilt-table can be used to provide a strong training stimulus to complement conventional physiotherapy practices and serve the dual purpose of increasing orthostatic tolerance and attenuating the decline in aerobic fitness [41].

Although MMSE and muscle strength of the upper limb in both the groups improved equally, the other parameters improved more after tilt-table exercises. It has been suggested that verticalization may play a role in stimulating cortical areas involved in the trunk and lower limb control, so that differentiation and learned non-use can be contrasted [42]. Verticalization may actively contribute to enhancing cognitive performance through an increase in cerebral blood flow with consequent induction of cortical plasticity, especially in frontal lobes [43]. Robotic verticalization includes increased ventilation, increased arousal, improved weight-bearing of the lower limbs, and facilitation of antigravity exercise of the limbs [44].

Thus, Erigo tilt- table exercises can be practiced for the rehabilitation of acute stroke patients where early mobilization is difficult and delayed due to trunk instability, orthostatic hypotension, reduce vigilance and cooperation.

Although the study found beneficial effects many respects, it has several limitations such as lack of long duration follow-up and a larger sample size which can be answered in future studies.

## Conclusion

The study concluded that both the Erigo tilt-table and conventional physiotherapy can to be beneficial and effective in the rehabilitation of acute stroke patients. This study also found that the tilt-table exercises were more beneficial in improving QOL, NIHSS, and muscle strength of the lower limb. The practitioner should consider Erigo tilt-table exercise to reduce the risk of complications and reduce impairments in stroke patients.

## Data Availability

The datasets used and/or analysed during the current study are available from the corresponding author on reasonable request.
